# Host Cathelicidin Exacerbates Group B *Streptococcus* Urinary Tract Infection

**DOI:** 10.1128/mSphere.00932-19

**Published:** 2020-04-22

**Authors:** Kathryn A. Patras, Alison Coady, Priyanka Babu, Samuel R. Shing, Albert D. Ha, Emma Rooholfada, Stephanie L. Brandt, Matthew Geriak, Richard L. Gallo, Victor Nizet

**Affiliations:** a Department of Pediatrics, University of California, San Diego, La Jolla, California, USA; b Sharp Memorial Hospital, San Diego, California, USA; c Department of Dermatology, University of California, San Diego, La Jolla, California, USA; d Skaggs School of Pharmacy and Pharmaceutical Sciences, University of California, San Diego, La Jolla, California, USA; University of Nebraska Medical Center

**Keywords:** cathelicidin, group B *Streptococcus*, innate immunity, mast cell, urinary tract infection

## Abstract

Group B *Streptococcus* (GBS) causes frequent urinary tract infection (UTI) in susceptible populations, including individuals with type 2 diabetes and pregnant women; however, specific host factors responsible for increased GBS susceptibility in these populations are not well characterized. Here, we investigate cathelicidin, a cationic antimicrobial peptide, known to be critical for defense during UTI with uropathogenic Escherichia coli (UPEC). We observed a loss of antimicrobial activity of human and mouse cathelicidins against GBS and UPEC in synthetic urine and no evidence for increased cathelicidin resistance in GBS urinary isolates. Furthermore, we found that GBS degrades cathelicidin in a protease-dependent manner. Surprisingly, in a UTI model, cathelicidin-deficient (*Camp*^−/−^) mice showed decreased GBS burdens and mast cell recruitment in the bladder compared to levels in wild-type (WT) mice. Pharmacologic inhibition of mast cells reduced GBS burdens and histamine release in WT but not *Camp*^−/−^ mice. Streptozotocin-induced diabetic mice had increased bladder cathelicidin production and mast cell recruitment at 24 h postinfection with GBS compared to levels in nondiabetic controls. We propose that cathelicidin is an important immune regulator but ineffective antimicrobial peptide against GBS in urine. Combined, our findings may in part explain the increased frequency of GBS UTI in diabetic and pregnant individuals.

**IMPORTANCE** Certain populations such as diabetic individuals are at increased risk for developing urinary tract infections (UTI), although the underlying reasons for this susceptibility are not fully known. Additionally, diabetics are more likely to become infected with certain types of bacteria, such as group B *Streptococcus* (GBS). In this study, we find that an antimicrobial peptide called cathelicidin, which is thought to protect the bladder from infection, is ineffective in controlling GBS and alters the type of immune cells that migrate to the bladder during infection. Using a mouse model of diabetes, we observe that diabetic mice are more susceptible to GBS infection even though they also have more infiltrating immune cells and increased production of cathelicidin. Taken together, our findings identify this antimicrobial peptide as a potential contributor to increased susceptibility of diabetic individuals to GBS UTI.

## Abstract (precis)

Certain populations such as diabetic individuals are at increased risk for developing urinary tract infections (UTI), although the underlying reasons for this susceptibility are not fully known. Additionally, diabetics are more likely to become infected with certain types of bacteria, such as group B *Streptococcus* (GBS). In this study, we find that an antimicrobial peptide called cathelicidin, which is thought to protect the bladder from infection, is ineffective in controlling GBS and alters the type of immune cells that migrate to the bladder during infection. Using a mouse model of diabetes, we observe that diabetic mice are more susceptible to GBS infection even though they also have more infiltrating immune cells and increased production of cathelicidin. Taken together, our findings identify this antimicrobial peptide as a potential contributor to increased susceptibility of diabetic individuals to GBS UTI.

## INTRODUCTION

Urinary tract infection (UTI) afflicts more than half of women at least once in their lifetime and generates over $2.4 billion in health care costs annually in the United States (1, 2). Acute UTI is typically mild, limited to the bladder (cystitis), and readily treated in healthy adults, but individuals with underlying metabolic and/or immune dysfunction such as type 2 diabetes and gestational diabetes are at risk for complications, including recurrent UTI, ascending infection (pyelonephritis), and urosepsis (3–5). While uropathogenic Escherichia coli (UPEC) is the predominant organism in UTI, group B *Streptococcus* (GBS) accounts for 1 to 2% of UTIs (6, 7), and increased GBS incidence in diabetic individuals has been reported in some cohorts (8) but not others (7, 9). Diabetes is associated with increased GBS asymptomatic bacteriuria (10) and is a leading risk factor for progression to invasive GBS disease (11–14). While the underlying molecular pathways are not understood, this clinical phenomenon implies that the urinary microenvironment may be altered to favor GBS colonization and dissemination in diabetes.

Systematic review and meta-analyses indicate that GBS asymptomatically colonizes 18% of pregnant women globally (15), and individual studies support similar colonization rates among other populations (16, 17). GBS is widely recognized as an agent of maternal infection and neonatal sepsis, and the highest incidence of GBS disease occurs in the first week of life (18). Heavy GBS vaginal carriage or GBS bacteriuria is associated with increased risk of preterm delivery and neonatal sepsis (19, 20). Current recommendations for universal screening of pregnant women to guide intrapartum antibiotic prophylaxis have reduced neonatal infections (18). However, 90% of GBS disease occurs outside the perinatal period, and infections, including UTI, of both adult and elderly populations are on the rise (12, 13, 18). Overall, GBS causes ∼160,000 UTIs annually in the United States (21), which is perhaps an underestimation based on recent improvements in culture techniques (22). GBS likely gains access to the female urinary tract through vaginal colonization, and a higher prevalence of GBS colonization is found in women with gestational diabetes (23–25), while the contribution of diabetes to GBS urogenital colonization outside pregnancy has not been extensively examined (26, 27). Effective host immune responses controlling GBS UTI are poorly characterized, and urinary immune suppression by the bacterium has been reported (28–30).

Multiple immune cells infiltrate the bladder during UTI, including neutrophils, monocytes, macrophages, dendritic cells, mast cells, CD4^+^ T cells, and NK cells (31–34). Additionally, multiple host factors protect against UTI through direct or indirect antibacterial activity, including antimicrobial peptides (AMPs) such as cathelicidins and β-defensins, iron-binding proteins, and soluble proteins that inhibit bacterial attachment (35–38). Many of these defense factors are subject to metabolic and hormonal regulation changes that occur during diabetes or pregnancy (39–42). Additionally, recent studies suggest that some, if not all, of these factors play immunomodulatory roles in the bladder (43–45).

Cathelicidins, a family of cationic AMPs, are produced by immune and epithelial cells and protect the host in part through direct killing of bacterial pathogens (46). Humans and mice each produce only one cathelicidin: LL-37 (encoded by *CAMP*) and CRAMP (encoded by *Camp*), respectively. Urinary cathelicidin levels increase dramatically during UTI such that cathelicidin is a suitable UTI biomarker (47). Resistance to cathelicidin killing is a hallmark of invasive UPEC isolates, and cathelicidin-deficient animals (*Camp*^−/−^) display increased bacterial burdens and tissue damage in some models (46) but not others (48), suggesting a complex role of cathelicidin in urinary tract protection. The bladder epithelium is primed to release cathelicidin and appears to be the primary source of the AMP during UTI (46). Cathelicidins are increasingly recognized as potent immunomodulators that influence monocyte and neutrophil chemotaxis, reactive oxygen species and cytokine production, and immune cell pattern recognition signaling (49–51). Exogenous administration of human cathelicidin LL-37 itself instigates cystitis in animal models (52, 53). Clinically, increased LL-37 levels are observed in GBS-susceptible populations, including diabetic patients (54, 55) and pregnant women (41). Additionally, the tissue microenvironment or *in vitro* medium testing conditions markedly influence cathelicidin antimicrobial activity (56–58).

Here, we investigate the role of cathelicidin in the host response to GBS UTI. We observe a loss of cathelicidin antimicrobial activity against GBS in urine and no selection for cathelicidin resistance across GBS urinary isolates. Rather, we find that cathelicidin-deficient mice are better able to control GBS bladder burdens than wild-type (WT) mice, in combination with reduced mast cell recruitment and degranulation. Using pharmacologic inhibition of mast cells and a streptozotocin (STZ)-induced diabetic murine model, we further link mast cell recruitment with an inability to control GBS bladder burdens. Combined, our findings may in part explain the increased frequency of GBS UTI in diabetic and pregnant individuals while highlighting mast cells as a potential therapeutic target in these patients.

## RESULTS

### Susceptibility to cathelicidin is similar for UPEC and GBS clinical isolates.

**FIG 1:**
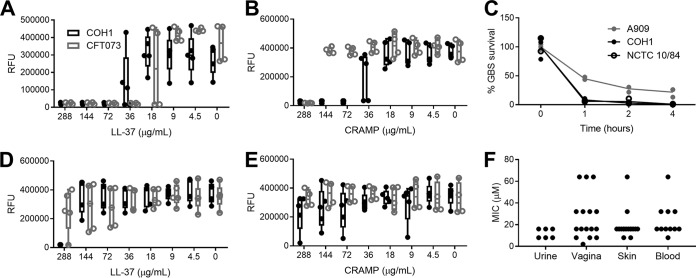
Susceptibility to cathelicidin is similar between UPEC and GBS clinical isolates. (A and B) MIC assays for COH1 and CFT073 using human LL-37 or murine CRAMP) in RPMI 1640 medium. (C) Killing kinetics of three GBS strains by 36 μg/ml (8 μM) LL-37 in RPMI 1640 medium over time. (D and E) MIC assays for COH1 and CFT073 using human LL-37 or murine CRAMP in synthetic urine. Symbols represent the means of independent experimental replicates (*n* = 3 to 5/group), with lines indicating means and SEM. (F) MIC values of GBS clinical isolates collected from urine, vagina, skin, or blood. Symbols represent the means of three independent experimental replicates (*n* = 6 to 16/group). Data were analyzed by Kruskal-Wallis with Dunn’s multiple-comparison test. RFU, relative fluorescence units.

To compare the susceptibility of GBS and UPEC to cathelicidin, MIC assays were performed with the widely studied GBS strain COH1, a hypervirulent sequence type 17 serotype III isolate, and UPEC strain CFT073, a pyelonephritis isolate. Bacteria were incubated with synthetic peptides for human LL-37 and murine CRAMP in tissue culture medium (RPMI 1640 medium) or synthetic urine (59) for 24 h. Due to lower optical densities of cultures grown in synthetic urine, MICs were determined by a >90% reduction in fluorescent signal from the conversion of resazurin to fluorescent resorufin, a sensitive readout of bacterial metabolic activity (60, 61). In RPMI 1640 medium, LL-37 MICs for GBS COH1 and UPEC CFT073 were 72 μg/ml (16 μM) and 36 μg/ml (8 μM), respectively, while CRAMP MICs were 72 μg/ml and 288 μg/ml (64 μM), respectively (Fig. 1A and B). Similar reductions in viable CFU counts were seen when GBS strains COH1, A909 (serotype Ia), and NCTC 10/84 (serotype V) were incubated with 36 μg/ml LL-37 in a 4-h kinetic killing assay (Fig. 1C). In contrast to results obtained in RPMI 1640 medium, GBS exposed to cathelicidin in synthetic urine was much more resistant to killing; the LL-37 MIC increased to 288 μg/ml for GBS COH1, with no growth inhibition of UPEC CFT073 at this concentration. Murine CRAMP did not inhibit either GBS or UPEC in synthetic urine at the highest concentration tested (288 μg/ml) (Fig. 1D and E). To assess broad patterns of GBS sensitivity to LL-37, 49 GBS clinical isolates from both colonizing (vaginal) and invasive (skin, blood, and urine) sites were subjected to LL-37 MIC testing in RPMI 1640 medium. The most common MIC for GBS isolates was 72 μg/ml (26 isolates), followed by 36 μg/ml (9 isolates) and 144 μg/ml (8 isolates). Across body sites, no significant differences in MICs were observed by a Kruskal-Wallis test (*P = *0.15) (Fig. 1F). Thus, while GBS and UPEC are similarly sensitive to cathelicidin in cell culture medium that reflects the ionic conditions of blood, conditions mimicking urine reduce cathelicidin antimicrobial activity. Furthermore, these data do not indicate a selective pressure for increased cathelicidin resistance among GBS urinary isolates compared to that of isolates from other body sites.

### GBS limits cathelicidin levels during bladder infection and exhibits cathelicidin proteolytic activity.

**FIG 2:**
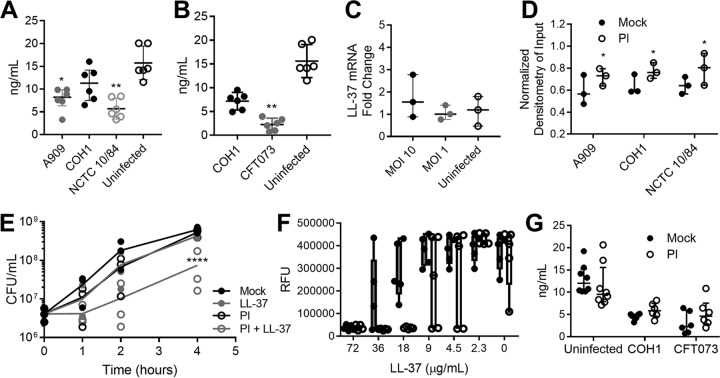
GBS infection limits bladder epithelium cathelicidin production, and GBS proteases degrade cathelicidin. (A and B) Human bladder epithelial cells (HTB-9) were infected with GBS A909, COH1, or NCTC 10/84 or with GBS COH1 and UPEC CFT073, as indicated, for 4 h at an MOI of 10. LL-37 production was measured by ELISA. (C) HTB-9 cells were infected with GBS COH1 for 3 h at an MOI of 10 or 1. LL-37 mRNA transcripts were quantified by qPCR and normalized to the level of the housekeeping gene, GAPDH, using the ΔΔ*C_T_* method. Symbols represent the means of independent experimental replicates (*n* = 3 to 6/group), with lines indicating medians and interquartile ranges. (D) GBS strains A909, COH1, and NCTC 10/84 were incubated with 9 μg/ml (2 μM) LL-37 for 4 h with or without addition of protease inhibitors (PI). Samples were spotted onto a nitrocellulose membrane and probed for LL-37. Densitometry was normalized to the signal from the 9-μg/ml LL-37 input. Raw images of dot blots are depicted in Fig. S1 in the supplemental material. (E) Susceptibility of GBS COH1 to 27 μg/ml (6 μM) LL-37 with or without protease inhibitors (PI) was measured over 4 h by serial dilution and plating. Symbols represent the means of three independent experiments, and lines indicate the mean values of experimental replicates. (F) MIC assay of GBS COH1 using human LL-37 in RPMI 1640 medium with (white bars) or without (gray bars) addition of protease inhibitors. Symbols represent the means of independent experimental replicates (*n* = 5/group), with lines indicating means and SEM. (G) Human bladder epithelial cells (HTB-9) were infected with GBS COH1 and UPEC CFT073 for 4 h at an MOI of 10 with or without addition of protease inhibitors. LL-37 production was measured by ELISA. Symbols represent the means of independent experimental replicates (*n* = 6 to 8/group), with lines indicating medians and interquartile ranges. Data were analyzed by Friedman’s test with Dunn’s multiple-comparison test (panels A to C), two-way repeated-measures ANOVA with Dunnett’s multiple-comparison test (panel E), or two-way ANOVA with Sidak’s multiple-comparison test (panels D and G). ****, *P < *0.0001; **, *P < *0.01; *, *P < *0.05.

To investigate how GBS infection influences cathelicidin in bladder epithelium, the human bladder epithelial cell line HTB-9 was infected with GBS strain A909, COH1, or NCTC 10/84 for 4 h at a multiplicity of infection (MOI) of 10 (10 bacteria to 1 host cell). Cells were lysed, and total LL-37 was quantified by enzyme-linked immunosorbent assay (ELISA). Interestingly, HTB-9 cells generated less LL-37 when infected with GBS strain A909 (mean, 7.8 ng/ml; *P = *0.042), COH1 (mean, 9.5 ng/ml; *P = *0.18), or NCTC 10/84 (mean, 5.7 ng/ml; *P = *0.0024) than the uninfected controls (mean, 15.7 ng/ml) (Fig. 2A). This reduction in LL-37 production was not restricted to GBS infection as HTB-9 cells infected with CFT073 for 4 h at an MOI of 10 also displayed lower LL-37 levels (mean, 2.2 ng/ml; *P = *0.0016) than COH1-infected (7.2 ng/ml) and uninfected controls (15.6 ng/ml) (Fig. 2B). To assess whether GBS infection impacted LL-37 transcription, HTB-9 cells were infected with COH1 for 3 h at an MOI of 10 or 1, and mRNA was quantified by quantitative PCR (qPCR) using glyceraldehyde-3-phosphate dehydrogenase (GAPDH) as a housekeeping gene. No significant differences were observed in LL-37 transcripts infected with GBS compared to results in uninfected controls (Fig. 2C). We used a lactate dehydrogenase (LDH) release assay to examine the impact of GBS and UPEC infection on HTB-9 cell viability, with data normalized to 1% Triton-X treatment (assumed 100% lysis). At an MOI of 1, toxicity was fairly low (<20% lysis) for all strains except GBS NCTC 10/84 (>70% lysis), a hyperhemolytic strain (see Fig. S1 in the supplemental material) (62). At an MOI of 10, GBS strains A909 and NCTC 10/84 both exhibited high toxicity (>70% lysis), whereas CFT073 showed moderate toxicity (24% lysis), and GBS COH1 showed minimal toxicity (<5% lysis) (Fig. S1).

10.1128/mSphere.00932-19.1 FIG S1 Cell viability of HTB-9 cells as measured by LDH release. GBS strains A909, COH1, and NCTC 10/84 or UPEC CFT073 were incubated with HTB-9 cells for 4 h at MOIs of 1 and 10. Supernatant was collected, and LDH was measured to monitor cell lysis. Data were normalized to uninfected HTB-9 cells treated with 1% Triton X-100 (used as a positive control, assumed total lysis) and expressed as percent lysis of the positive control. Download FIG S1, TIF file, 0.2 MB. Copyright © 2020 Patras et al. 2020 Patras et al. This content is distributed under the terms of the Creative Commons Attribution 4.0 International license.

Cathelicidin is a target of degradation by bacterial proteases, as shown for Pseudomonas aeruginosa, Enterococcus faecalis, Proteus mirabilis, group A *Streptococcus*, and enteropathogenic E. coli (63–65). To determine if GBS degradation of cathelicidin was occurring, GBS strains A909, COH1, and NCTC 10/84 were incubated with 9 μg/ml (2 μM, sub-MIC level) of LL-37 for 4 h with or without the addition of a protease inhibitor (PI) cocktail. Dot blot analysis revealed degradation of LL-37 across GBS strains, with a partial rescue of LL-37 levels in the presence of a protease inhibitors (Fig. 2D, densitometry analysis; see uncropped blots in Fig. S2A to C). Similar degradation of murine CRAMP was observed across GBS strains (Fig. S2D). Addition of a protease inhibitor in the presence of 27 μg/ml (6 μM) LL-37 significantly reduced COH1 survival at 4 h (mean, 4.3e8 CFU/ml; *P* ≤ 0.0001) compared to that of LL-37-treated COH1 cells without protease inhibitors (7.4e7 CFU/ml) (Fig. 2E). Additionally, protease inhibition reduced the LL-37 MIC from 72 μg/ml to 18 μg/ml (4 μM) (Fig. 2F). Protease activity alone did not fully account for the reduction of LL-37 in GBS- and UPEC-infected cells as protease inhibitor treatment did not significantly rescue LL-37 production during infection with COH1 (mean, 5.9 ng/ml for COH1 with PI; mean 4.6 ng/ml COH1 infection alone) or CFT073 (mean, 3.1 ng/ml CFT073 with PI; mean, 5.3 ng/ml for CFT073 infection alone) compared to that in uninfected controls (PI treatment mean, 1.3 ng/ml; no-treatment mean, 13.0 ng/ml) (Fig. 2G). Specific protease inhibitors for serine, cysteine, aspartyl, and aminopeptidase proteases were also added to GBS cultures with or without 27 μg/ml LL-37; no single protease inhibitor achieved the level of reduced GBS survival equal to that of the protease inhibitor cocktail (Fig. S3).

10.1128/mSphere.00932-19.2 FIG S2 Raw images of dot blots probed for LL-37 and CRAMP. GBS strains A909, COH1, and NCTC 10/84 were incubated with 9 μg/ml (2 μM) LL-37 (A to C) or CRAMP (D) for 4 h with or without addition of protease inhibitors (PI). Samples were spotted onto a nitrocellulose membrane and probed for LL-37 and CRAMP, respectively, as described in Materials and Methods. Download FIG S2, TIF file, 2.0 MB. Copyright © 2020 Patras et al. 2020 Patras et al. This content is distributed under the terms of the Creative Commons Attribution 4.0 International license.

10.1128/mSphere.00932-19.3 FIG S3 Use of specific protease inhibitors fails to identify the specific GBS protease(s) that degrades cathelicidin. (A and B) Susceptibility of GBS COH1 to 27 μg/ml (6 μM) LL-37 with or without protease inhibitors (PI) as indicated in panel legends and detailed in Materials and Methods. Viable GBS was measured over 4 h by serial dilution and plating. Symbols represent the means of three independent experiments, and error bars indicate SEM. Download FIG S3, TIF file, 0.2 MB. Copyright © 2020 Patras et al. 2020 Patras et al. This content is distributed under the terms of the Creative Commons Attribution 4.0 International license.

### Cathelicidin deficiency reduces GBS burden in the bladder.

**FIG 3:**
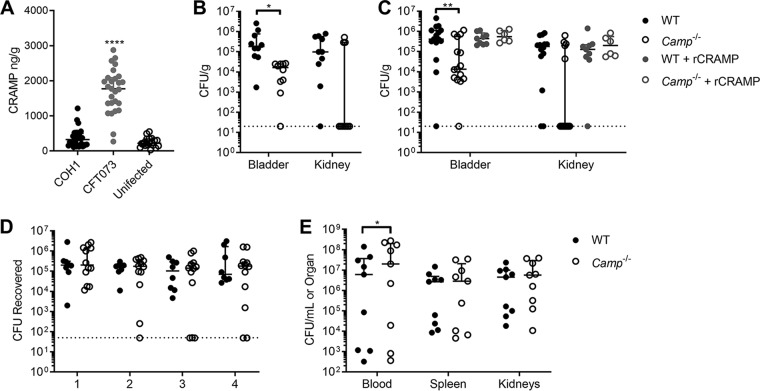
Cathelicidin deficiency reduces GBS burden in the bladder. (A) WT C57BL/6 female mice were transurethrally infected with 2 × 10^7^ CFU of GBS COH1 or UPEC CFT073 or mock infected as a control. Bladders were collected at 24 h postinfection, and cathelicidin levels were quantified by ELISA. (B) WT C57BL/6 and *Camp*^−/−^ female mice, as indicated, were transurethrally infected with 2 × 10^7^ CFU of GBS COH1. Bladders and kidneys were collected at 24 h postinfection to determine GBS burdens. (C) WT C57BL/6 and *Camp*^−/−^ mice were infected with GBS COH1 as described for panel B, with the inclusion of recombinant CRAMP treatment (320 μM) transurethrally 1 h prior to infection or mock treatment with PBS as a control. Bladders and kidneys were collected at 24 h postinfection to determine GBS burdens. (D) WT C57BL/6 and *Camp*^−/−^ female mice were vaginally administered 2 × 10^7^ CFU of GBS COH1. Mice were vaginally swabbed daily, and the levels of GBS CFU recovered from swabs are shown. (E) WT C57BL/6 and *Camp*^−/−^ male mice were injected i.p. with 2 × 10^7^ CFU of GBS COH1. Blood, spleen, and kidneys were collected at 24 h postinfection to determine GBS burdens. Experiments were conducted at least two times independently, and data were combined. Symbols represent biological replicates (*n* = 8 to 32/group), with lines indicating medians with interquartile ranges. Dotted lines indicate limits of detection for CFU. Data were analyzed using Kruskal-Wallis with Dunn’s multiple-comparison test (panel A) or two-way repeated measures ANOVA with Sidak’s multiple-comparison test (panels B to E). ****, *P < *0.0001; **, *P < *0.01; *, *P < *0.05.

Since cathelicidin itself is a biomarker for UTI (47), we studied its production during UTI in a murine model. WT C57BL/6 female mice were infected with GBS COH1 or UPEC CFT073 or mock infected with phosphate-buffered saline (PBS). Bladders were collected at 24 h postinfection, and cathelicidin was quantified by ELISA. Mice infected with UPEC CFT073 had robust induction of cathelicidin (median, 1,756 ng/g; *P < *0.0001) compared to that of mock-infected controls (median, 220 ng/g), whereas mice infected with GBS lacked this robust increase (median, 327 ng/g; *P = *0.48) (Fig. 3A). To test the biological relevance of cathelicidin during GBS UTI, WT and cathelicidin-deficient *Camp*^−/−^ mice were infected with GBS COH1, and bladders and kidneys were collected at 24 h postinfection. Surprisingly, *Camp*^−/−^ mice displayed lower GBS burdens (median, 1.63e4 CFU/g; *P = *0.026) in the bladder than WT mice (median, 1.82e5 CFU/g) (Fig. 3B). No significant differences in burdens were observed in the kidneys between WT and *Camp*^−/−^ mice (median, 9.76e4 CFU/g and not detected, respectively; *P = *0.69).

To determine if this phenotype correlated with an acute deficiency of cathelicidin during infection, rather than a sustained effect of cathelicidin knockout (KO) on bladder and/or immune development and maturation, WT and *Camp*^−/−^ mice were instilled with a single intravesicular dose of recombinant CRAMP (rCRAMP; 320 μM) 1 h prior to GBS infection. At 24 h postinfection, pretreatment with rCRAMP resolved differences in bladder burdens between WT and *Camp*^−/−^ mice (median, 4.41e5 CFU/g and 5.47e5 CFU/g, respectively; *P = *0.96), whereas untreated mice recapitulated differences seen in the data shown in Fig. 3B (median, 4.12e5 CFU/g and 1.35e4 CFU/g, respectively; *P = *0.0071) (Fig. 3C). Treatment with rCRAMP did not significantly increase GBS bladder burdens over the levels in mock-treated mice. Additionally, no significant differences were observed in kidney burdens across genotypes and treatment groups (Fig. 3C).

To assess the contribution of cathelicidin to GBS-host interactions in additional relevant models, we utilized murine models of vaginal colonization and sepsis. No differences in GBS persistence were observed during vaginal colonization between WT and *Camp*^−/−^ mice (Fig. 3D). Furthermore, in a sepsis model of GBS, *Camp*^−/−^ mice displayed a modest increase in blood bacterial load compared to that of WT mice (2.0e7 CFU/ml and 6.0e6 CFU/ml, respectively; *P = *0.035) but no differences in spleen or kidney burdens (Fig. 3E). Together, these data suggest that the enhanced resistance of *Camp*^−/−^ mice to GBS in the bladder is specific to UTI and the bladder microenvironment.

### Cathelicidin deficiency alters bladder immune cell populations in response to GBS.

**FIG 4:**
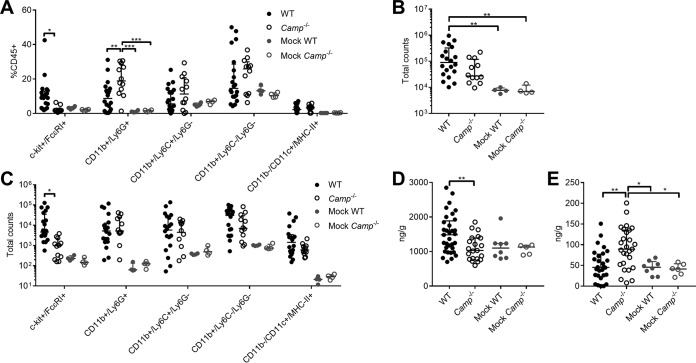
Cathelicidin deficiency alters bladder immune cell populations in response to GBS. WT C57BL/6 and *Camp*^−/−^ female mice were transurethrally infected with 2 × 10^7^ CFU of GBS COH1 or mock infected as a control. Bladders were collected at 24 h postinfection and analyzed via flow cytometry The gating strategy of CD45^+^ lymphocytes is described in Fig. S4 in the supplemental material. (A) Percentage of CD45^+^ cells staining for given surface markers. (B) Total counts of CD45^+^ cells from mouse bladders. (C) Total counts of cells from panel B which stained for given surface markers. Bladders were also subjected to histamine (D) and myeloperoxidase (MPO) ELISA (E). Experiments were conducted at least two times independently, and data were combined. Symbols represent biological replicates (*n* = 4 to 33/group), with lines indicating medians with interquartile ranges. Data were analyzed using two-way repeated-measures ANOVA with Tukey’s multiple-comparison test (panels A and C), Kruskal-Wallis with Dunn’s multiple-comparison test (panel B), and one-way ANOVA with Holm-Sidak’s multiple-comparison test (panels D and E). Significant differences are shown. All other comparisons are not significant (*P > *0.05). ***, *P < *0.001; **, *P < *0.01; *, *P < *0.05.

Based on the observations of poor antimicrobial activity of cathelicidin in urine and lower GBS burdens in *Camp*^−/−^ mice, we characterized the immune cell profiles of WT and *Camp*^−/−^ mice during GBS UTI using flow cytometry. Bladder cells were collected from WT and *Camp*^−/−^ mice at 24 h postinfection with GBS and stained with antibodies for the following cell surface markers: CD45, CD11b, CD11c, majory histocompatibility complex class II (MHC-II), Ly6C, Ly6G, FcεRI, and c-kit. See Fig. S4 for the gating strategy. Similar to previous findings on immune cell populations in the bladder during UPEC infection (31, 66), dominant CD45^+^ populations 24 h postinfection consisted of neutrophils (CD11b^+^ CD11c^+/−^ Ly6G^+^), macrophages/NK cells (CD11b^+^ CD11c^+/−^ Ly6C^−^ Ly6G^−^), monocytes (CD11b^+^ CD11c^+/−^ Ly6C^+^), mast cells (c-kit^+^ FcεRI^+^), and antigen-presenting cells (CD11c^+^ MHC-II^+^) (^+/−^ annotation indicates cells in that category may be either positive or negative for staining of CD11c). While no differences were seen in immune cell populations in mock-infected animals, infected WT mice displayed higher mast cell percentages than *Camp*^−/−^ mice (median, 10.6% and 2.0% of CD45^+^, respectively; *P = *0.021). Furthermore, infected *Camp*^−/−^ mice exhibited higher percentages of neutrophils than infected WT mice (median, 18.9% and 8.7% of CD45^+^, respectively; *P = *0.0046) (Fig. 4A). While total CD45^+^ cells increased in WT infected mice compared to the level in mock-infected mice, there was no significant increase in the level in *Camp*^−/−^-infected mice over that in mock-infected mice (Fig. 4B). Importantly, WT infected mice displayed significantly more mast cells than *Camp*^−/−^ mice (median, 4,014 cells and 1,038 cells per bladder, respectively; *P = *0.032), but no significant increase in neutrophil counts was observed (Fig. 4C).

10.1128/mSphere.00932-19.4 FIG S4 Gating scheme for flow cytometry. Cells were first gated for lymphocyte populations based on side scatter versus forward scatter area (SSC-A versus FSC-A, respectively), followed by gating for singlets (FSC height [FSC-H] versus FSC-A). The lymphocyte gate was further analyzed by expression of CD45. CD45^+^ cells were assessed for CD11b and CD11c surface markers. CD11b^+^ CD11c^+/−^ cells were considered myeloid lineage cells. Antigen-presenting cells were identified as CD11b^−^ CD11c^+^ MHC-II^+^, mast cells were identified as CD11b^−^ CD11c^−^ c-kit^+^ FcεRI^+^, macrophages/NK cells were considered CD11b^+^ CD11c^+/−^ Ly6G^−^ Ly6C^−^, monocytes were considered CD11b^+^ CD11c^+/−^ Ly6G^−^ Ly6C^+^, and neutrophils were considered CD11b^+^ CD11c^+/−^ Ly6G^+^ Ly6C^−^. Download FIG S4, TIF file, 0.5 MB. Copyright © 2020 Patras et al. 2020 Patras et al. This content is distributed under the terms of the Creative Commons Attribution 4.0 International license.

Mast cells are highly granulated innate immune cells that serve multiple functions, including surveillance of pathogens through Toll-like receptor (TLR) signaling, proinflammation via histamine release, and direct bacterial killing through cathelicidin and protease release (67). Similarly, neutrophils are vital to antibacterial defense of the bladder, as demonstrated through multiple neutrophil depletion studies (32, 68, 69). To corroborate flow cytometry counts, bladders from GBS-infected and mock-infected WT and *Camp*^−/−^ mice were collected at 24 h postinfection and subjected to ELISA for histamine and neutrophil myeloperoxidase (MPO). WT infected mice exhibited increased levels of histamine compared to those in *Camp*^−/−^ infected mice (median, 1,484 ng/g and 1,031 ng/g, respectively; *P = *0.0078) (Fig. 4D). In infected animals, MPO levels were increased in *Camp*^−/−^ mice over levels in WT mice (median, 89.2 ng/g and 45.2 ng/g, respectively; *P = *0.0020) (Fig. 4E). No differences in either histamine or MPO levels between mock-infected WT and *Camp*^−/−^ mice were observed.

### Mast cell inhibitor cromolyn sodium reduces GBS burden in the bladder.

**FIG 5:**
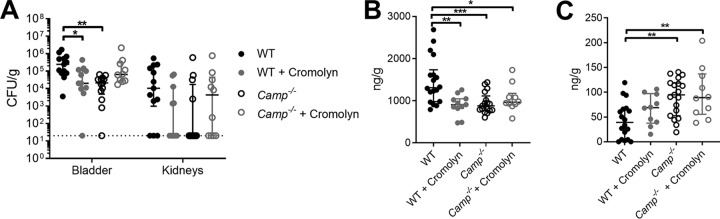
Mast cell inhibitor cromolyn sodium reduces GBS burden in the bladder. WT C57BL/6 and *Camp*^−/−^ female mice were treated with the mast cell membrane stabilizer cromolyn sodium at 10 mg/kg/dose at 48 h, 24 h, and 1 h prior to transurethral infection with 2 × 10^7^ CFU of GBS COH1. Mock-treated mice were used as controls. Bladder and kidneys were collected at 24 h postinfection to determine bacterial burdens (A). Bladders were also subjected to histamine (B) and myeloperoxidase (MPO) ELISA (C). Experiments were conducted at least two times independently, and data were combined. Symbols represent biological replicates (*n* = 10 to 18/group), with lines indicating medians with interquartile ranges. Data were analyzed using two-way repeated-measures ANOVA with Tukey’s multiple-comparison test (panel A) and one-way ANOVA with Tukey’s multiple-comparison test (panels B and C). ***, *P < *0.001; **, *P < *0.01; *, *P < *0.05.

Because WT mice infected with GBS COH1 displayed both increased bacterial burdens and mast cell numbers in the bladder at 24 h postinfection, we next assessed if mast cell inhibition might improve host outcomes during GBS UTI. Mast cell degranulation can be pharmacologically inhibited using the FDA-approved drug cromolyn sodium, previously shown to be efficacious in reducing bladder inflammation in mice (70). WT and *Camp*^−/−^ mice were treated with 10 mg/kg of cromolyn sodium injected intraperitoneally (i.p.) at 48 h, 24 h, and 1 h prior to bladder infection with COH1. WT mice receiving cromolyn exhibited a significant reduction in GBS bladder burden at 24 h postinfection compared to levels in mock-treated WT mice (median, 2.01e4 CFU/g and 2.47e5 CFU/g, respectively; *P = *0.043) (Fig. 5A). *Camp*^−/−^ mice receiving cromolyn had GBS burdens similar to those of mock-treated *Camp*^−/−^ mice (6.21e4 CFU/g and 2.09e4 CFU/g, respectively; *P = *0.12). As seen in Fig. 3B and C, mock-treated WT mice had significantly higher GBS burdens than mock-treated *Camp*^−/−^ mice (*P = *0.0078). No significant differences in kidneys of GBS-infected mice were observed among all groups (Fig. 5A). To determine whether cromolyn treatment reduced mast cell degranulation in the bladders of WT mice, bladder homogenates were subjected to ELISA for histamine and neutrophil myeloperoxidase (MPO). Cromolyn treatment significantly reduced histamine levels in WT mice compared to levels in mock-treated WT mice (median, 905 ng/g and 1,292 ng/g, respectively; *P = *0.0015) (Fig. 5B) whereas cromolyn treatment did not alter histamine levels in *Camp*^−/−^ mice (961 ng/g and 877 ng/g, respectively; *P > *0.99). As seen in the data of Fig. 4D, WT mock-treated mice displayed higher histamine levels than mock-treated *Camp*^−/−^ mice (*P = *0.0009) and cromolyn-treated *Camp*^−/−^ mice (*P = *0.042). Conversely, cromolyn treatment did not impact levels of MPO in WT mice (median, 67.9 ng/g in cromolyn-treated mice and 39.1 ng/g in mock-treated mice; *P > *0.5057) (Fig. 5C). Similar to the data shown in Fig. 4E, both mock-treated *Camp*^−/−^ (median, 95.5 ng/g) and cromolyn-treated *Camp*^−/−^ (median, 89.2 ng/g) mice had higher MPO levels than WT mice (*P = *0.0033 and *P = *0.0044, respectively) (Fig. 5C).

Since mast cells are protective in a variety of Gram-positive and Gram-negative bacterial infections (71), we tested whether mast cells controlled and/or killed GBS *in vitro*. The human mast cell line HMC-1 was infected with GBS COH1 at an MOI of 0.1 for 60 min, and total counts of recovered bacteria were determined by serial dilution and plating. HMC-1 cells failed to control GBS growth (median recovered CFU amount, 122% of inoculum) (Fig. S5A). Furthermore, a 30-min pretreatment of HMC-1 with 100 μM cromolyn sodium (72), physiologic levels of LL-37 (288 ng/ml), or both cromolyn and LL-37 combined did not alter the amount of GBS CFU recovered (Fig. S5A). Similarly, HMC-1 cells infected with UPEC CFT073 failed to demonstrate potent bacterial killing (median recovered CFU amount, 98.4% of inoculum) (Fig. S5B), nor did cromolyn sodium or LL-37 treatment alter amounts of UPEC CFU recovered (Fig. S5B).

10.1128/mSphere.00932-19.5 FIG S5 Total mast cell counts are increased *in vivo*, but no GBS killing by mast cell lines was observed *in vitro*. Human mast cell line HMC-1 was infected with GBS COH1 (A) or UPEC CFT073 (B) at an MOI of 0.1 for 60 min. HMC-1 cells were pretreated with 100 μM cromolyn sodium for 30 min, and/or infection was done in the presence of 288 ng/ml (64 nM) LL-37 where indicated. Percent killing was calculated as a percentage of CFU from wells containing bacteria only at the experimental end point. Symbols represent the mean of values of three independent experiments. Download FIG S5, TIF file, 0.3 MB. Copyright © 2020 Patras et al. 2020 Patras et al. This content is distributed under the terms of the Creative Commons Attribution 4.0 International license.

### STZ-induced diabetes increases susceptibility to GBS UTI.

**FIG 6:**
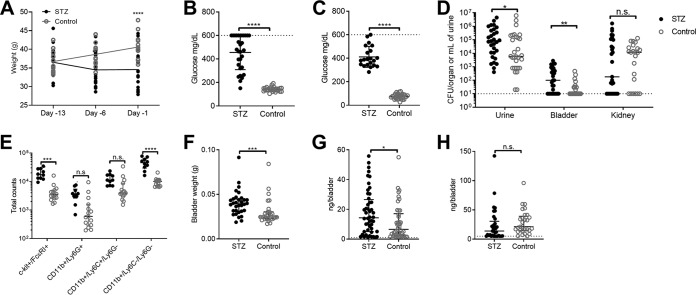
Streptozotocin-induced diabetes increases susceptibility to GBS UTI. WT CD1 female mice were treated with 4 doses of 80/mg/kg/dose streptozotocin (STZ) as described in Materials and Methods. Mock-treated mice were used as controls. At 3 weeks following treatment, mice were transurethrally infected with 2× 10^7^ CFU of GBS COH1. (A) Body weights were collected weekly following STZ treatment. (B) Blood glucose at 24 h prior to infection. (C) Urine glucose collected at 24 h postinfection. (D) Urine, bladder, and kidneys were collected at 24 h postinfection to determine bacterial burdens. (E) Bladders collected at 24 h postinfection were analyzed via flow cytometry. The gating strategy is described in Fig. S4 in the supplemental material. (F) Bladder weights of mice at 24 h postinfection. Bladders were subjected to cathelicidin ELISA (G) and histamine ELISA (H). Experiments were conducted at least two times independently, and data were combined. Symbols represent biological replicates (*n* = 10 to 30/group), with lines indicating medians with interquartile ranges. Dotted lines indicate upper limits of detection for glucose meter (panels B and C) and lower limits of detection for CFU (panel D) or ELISA (panels G and H). Data were analyzed by two-way repeated-measures ANOVA with Sidak’s multiple-comparison test (panels A, D, and E) and a two-tailed Mann-Whitney test (panels B, C, and F to H). ****, *P < *0.0001; ***, *P < *0.001; **, *P < *0.01; *, *P < *0.05; n.s., not significant.

Patients with type 2 diabetes or gestational diabetes are more susceptible to GBS invasive disease, including UTI and soft tissue infections (12, 13, 21), but the host factors contributing to GBS susceptibility are not well described. To investigate if an animal model of diabetes can recapitulate the enhanced GBS susceptibility observed in the human population, we utilized an inducible murine model of diabetes using the compound streptozotocin (STZ) (73). WT CD1 female mice received daily doses of 80 mg/kg STZ i.p. for 4 days or were mock treated as controls beginning 20 to 22 days prior to infection. At 1 week posttreatment, body weights were similar between STZ-treated and control mice (mean, 36.5 g versus 36.7g; *P = *0.99) (Fig. 6A). STZ-treated mice exhibited moderate weight loss approximately 2 weeks posttreatment (34.5 g versus 38.7 g; *P = *0.0054); however, weight loss was stabilized by 1 day prior to GBS infection (34.6 g versus 40.6 g; *P < *0.0001). Additionally, STZ-treated mice were hyperglycemic (mean, 393 mg/dl in STZ-treated mice versus 142 mg/dl in controls; *P < *0.0001) (Fig. 6B), with glucosuria (mean, 478 mg/dl in STZ-treated mice versus 96.5 mg/dl in controls; *P < *0.0001) (Fig. 6C) 24 h prior to infection. Mice were transurethrally infected with 2 × 10^7^ CFU GBS COH1, and at 24 h postinfection, urine, bladder, and kidneys were collected. STZ-treated mice had significantly higher GBS burdens in the urine than mock-treated control mice (median, 1.04e5 CFU/ml and 5.60e3 CFU/ml, respectively; *P = *0.046) and bladder (median, 1.33e2 CFU/organ and 1.5e2 CFU/organ, respectively; *P = *0.0057) (Fig. 6D). No significant differences were observed in the kidneys of STZ-treated and control mice (median, 1.87e2 CFU/organ and 1.41e4 CFU/organ, respectively; *P = *0.88).

To examine whether differences in bacterial burdens in STZ-treated mice impacted bladder immune cell populations, we performed flow cytometry of bladders at 24 h postinfection. STZ-treated mice showed no changes to infiltrating neutrophils (CD11b^+^ Ly6C^−^ Ly6G^+^) and monocytes (CD11b^+^ Ly6C^+^ Ly6G^−^) (Fig. 6E). However, STZ-treated mice displayed significantly higher populations of mast cells (c-kit^+^ FcεRI^+^) than control mice (1.75e4 cells/bladder and 3.51e3 cells/bladder, respectively; *P < *0.0001) (Fig. 6E). Additionally, significantly higher numbers of CD11b^+^ Ly6C^−^ Ly6G^−^ cells were observed in STZ-treated mice than in control mice (4.58e4 cells/bladder and 1.05e4 cells/bladder, respectively; *P < *0.0001) (Fig. 6E). Because bladder weights of STZ-treated mice (median, 0.0376 g) were higher than those of control mice (median, 0.0237 g; *P = *0.0002) (Fig. 6F), cathelicidin and histamine levels, measured by ELISA, were compared as total amounts detected per bladder. Bladder levels of cathelicidin were significantly higher in STZ-treated mice (median, 14.7 ng/bladder) than in control mice (median, 5.9 ng/bladder; *P = *0.030) (Fig. 6G). However, no differences were observed in histamine levels between STZ-treated mice (median, 13.9 ng/bladder) and control mice (median, 19.6 ng/bladder; *P = *0.053) (Fig. 6H).

**FIG 7:**
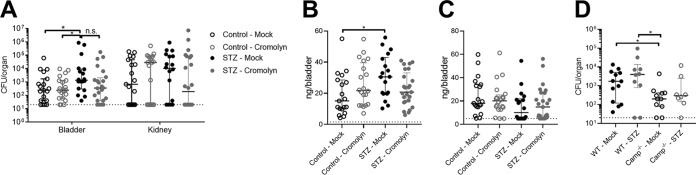
Impact of cromolyn sodium treatment and CRAMP deficiency in STZ-induced diabetic mice. WT CD1 female mice were treated with streptozotocin (STZ) and/or cromolyn sodium and infected with 2 × 10^7^ CFU of GBS COH1 as described in Materials and Methods. (A) Bladder and kidneys were collected at 24 h postinfection to determine bacterial burdens. Bladders were subjected to cathelicidin ELISA (B) and histamine ELISA (C). (D) WT C57BL/6 and *Camp*^−/−^ female mice were treated with STZ and infected with 2 × 10^7^ CFU of GBS COH1 as described in Materials and Methods, with bladders collected 24 h postinfection. Mock-treated animals from the experiments shown in Fig. 3B are shown for comparison. Experiments were conducted one to two times independently, and data were combined where appropriate. Symbols represent biological replicates (*n* = 8 to 25/group), with lines indicating medians with interquartile ranges. Dotted lines indicate lower limits of detection for CFU (panels A and D) or ELISA (panels B and C). Data were analyzed using two-way repeated-measures ANOVA with Tukey’s multiple-comparison test (panel A) and one-way ANOVA with Tukey’s multiple-comparison test (panels B to D). *, *P < *0.05; n.s., not significant.

Nonetheless, given the higher numbers of mast cells observed in STZ-treated mice (Fig. 6E), we examined whether the mast cell inhibitor cromolyn sodium would impact control of GBS burdens. Interestingly, although cromolyn sodium treatment did not significantly reduce GBS burdens in STZ-treated mice (*P = *0.0584), cromolyn sodium treatment resolved differences between control mice and STZ-treated mice (Fig. 7A). Additionally, STZ-treated mice given cromolyn sodium, unlike the mock-treated STZ-treated mice, did not show significantly higher cathelicidin levels (Fig. 7B). No significant changes in histamine levels were observed across groups (Fig. 7C). To examine whether cathelicidin deficiency was still protective in diabetes, WT and *Camp*^−/−^ mice were treated with STZ and infected with GBS, and bladder burdens were compared to those of nondiabetic mice (values from the experiment shown in Fig. 3B). WT mice treated with STZ demonstrated higher GBS burdens per bladder than nondiabetic WT mice (median, 3,800 CFU and 1,800 CFU, respectively; *P = *0.13), nondiabetic *Camp*^−/−^ mice (median, 200 CFU; *P = *0.018), and diabetic *Camp*^−/−^ mice (median, 260 CFU; *P = *0.15). Conversely, GBS burdens in bladders of STZ-treated *Camp*^−/−^ mice were similar to those of nondiabetic *Camp*^−/−^ mice (*P = *0.71) (Fig. 7D). Together, these findings support a model in which cathelicidin production in diabetic UTI correlates with an ineffective immune response and in which pharmacologic inhibition of mast cells may enhance host control of GBS.

## DISCUSSION

Individuals with dysfunctional metabolisms, such as those with type 2 diabetes and gestational diabetes, are at increased risk for UTI, with GBS disproportionately represented as a causative agent compared to causes of UTI in otherwise healthy individuals (10). Here, we investigated the antimicrobial peptide cathelicidin in host defense against GBS UTI. Unexpectedly and in contrast to a protective role in UPEC UTI (46), we found that cathelicidin aggravates GBS UTI in a manner dependent on mast cell degranulation. We propose that cathelicidin is an important immune regulator but ineffective antimicrobial peptide against GBS in urine, which may in part explain the increased frequency of GBS UTI in diabetic and pregnant individuals.

Although cathelicidin protects the urinary tract against UPEC infection, presumably through antimicrobial activity (46), we failed to detect antimicrobial activity of LL-37 or CRAMP against UPEC in synthetic urine at concentrations up to 288 μg/ml (Fig. 1). Similarly, we observed a loss of antimicrobial activity toward GBS in synthetic urine. Modest increased bacterial resistance to LL-37 and CRAMP has been previously reported under high-salt conditions (58, 74), and this may be one factor contributing to the loss of antimicrobial activity in urine. We observed that 27% (13/49) of GBS clinical isolates showed resistance to cathelicidin killing at a concentration of 32 μM or higher in tissue culture medium (Fig. 1), a level of resistance observed previously among GBS strains (75, 76). Additionally, we did not observe selective pressure for cathelicidin resistance in GBS urinary isolates, in contrast to previous observations with UPEC pyelonephritis isolates (46). Even so, the range of susceptibilities of GBS clinical isolates was similar to that previously reported for UPEC cystitis and pyelonephritis isolates (30 to 60 μM) (46). Similar to our findings with GBS, CRAMP-deficient mice were recently found to be more resistant to a UPEC cystitis isolate (48); however, unlike infection of *Camp*^−/−^ mice with GBS (Fig. 4), CRAMP deficiency in UPEC infection coincided with attenuated immune cell recruitment and decreased tissue damage. Collectively, these data support a more complex role for cathelicidins in the urinary tract beyond antimicrobial mechanisms.

Probing the reduction of LL-37 in GBS-infected bladder epithelial cells, we found that multiple GBS strains could degrade LL-37 in a protease-dependent manner and that GBS is further sensitized to LL-37 with the addition of protease inhibitors (Fig. 2). Proteolysis of cathelicidins has been reported by serine proteases, cysteine proteases, and metalloproteases from human-pathogenic bacteria (77–79), but testing with specific protease inhibitors did not allow us to pinpoint the type(s) of GBS protease responsible for this degradation (see Fig. S3 in the supplemental material). Of note, addition of protease inhibitors to bladder epithelial cells infected with either GBS or UPEC was not sufficient to rescue production of LL-37 compared to levels in uninfected cells (Fig. 2F). This observation is in line with reports by Chromek et al. (46), which demonstrated an initial surge in cathelicidin transcripts during UPEC infection but not later time points, suggesting that cell stress may be a factor limiting production of LL-37. Additionally, the HTB-9 bladder cell line was isolated from a grade II carcinoma and may not accurately reflect responses of the healthy or diabetic bladder epithelium.

Whether cathelicidin is an effective antimicrobial defense against GBS remains unclear, and its activity may be tissue dependent. In this study, addition of a single dose of rCRAMP 1 h prior to infection (72 μg), which was well above endogenous levels of CRAMP in UPEC-infected mice (maximum level detected, 150 ng/bladder), did not reduce GBS bladder burdens in WT or *Camp*^−/−^ mice (Fig. 3). In bovine mastitis, cathelicidin is strongly induced, particularly when GBS is the causative agent (80). In the reproductive tract, cathelicidin levels rise during pregnancy (81), but the amount of cathelicidin is insufficient to kill GBS (82). This observation is corroborated in our murine model as cathelicidin deficiency did not impact GBS vaginal colonization (Fig. 3D); however, we did not examine colonization beyond 4 days, so the possibility of a delayed phenotype cannot be ruled out. During sepsis, cathelicidin may confer partial protection in controlling GBS burden in the bloodstream though other organs, such as the spleen and kidneys, were not impacted by cathelicidin deficiency in our experiments. In the bladder, mice did not show robust induction of cathelicidin upon GBS infection, particularly compared with levels in UPEC-infected mice (Fig. 3A), but it is currently unknown whether this same phenomenon occurs in humans during GBS UTI. In one study, elevated plasma and urinary LL-37 levels were observed in patients with UTI; however, GBS was not a causative UTI agent in this particular cohort (47). Alternatively, it is possible that GBS actively suppresses cathelicidin production in the urogenital tract. In placental specimens, significantly lower cathelicidin placental levels were observed in GBS-positive women (83). However, in this study, we saw no evidence of alterations to bladder LL-37 transcription during GBS infection *in vitro* (Fig. 2C).

Cathelicidins are increasingly appreciated as immunomodulatory peptides that can activate or suppress inflammation and exert chemotactic effects on a variety of immune cells (84). Epithelial, resident, and invading innate immune cells can serve as sources for cathelicidin during infection (84). When we analyzed the immune profiles of cathelicidin-deficient mice in response to GBS UTI, we found a selective reduction in mast cell recruitment and degranulation (Fig. 4). Similarly, human LL-37 induces mast cell chemotaxis and degranulation and increases mast cell expression of TLR4 (85–87). Mast cell activation by LL-37 is thought to act through the MrgX2 receptor, a G protein-coupled receptor (GPCR), but other receptors, including FPRL1 and P2X7, have been proposed (88). The proinflammatory functions of cathelicidin have been observed in many human diseases and mouse inflammatory models (51, 89–92), and this capacity to enhance tissue inflammation is consistent with the observations in our study. Interestingly, in the absence of cathelicidin, we saw an enhancement of neutrophil MPO release (Fig. 4), which may explain the reduction in GBS CFU levels compared to those of WT mice, but the molecular factors promoting this neutrophil response are beyond the scope of this study.

Mast cells have been implicated in the host defense against numerous bacterial pathogens. In addition to supporting neutrophil and macrophage antimicrobial properties, mast cells themselves possess mechanisms to directly kill bacteria, including release of proteases, extracellular traps, and antimicrobial peptides, including cathelicidins (71, 93, 94). In the context of UPEC UTI, mast cells provide host protection. UPEC infection drives mast cell recruitment to the bladder and stimulates histamine release via FimH (34). Additionally, interleukin-1β (IL-1β) and/or inflammasome activation triggers mast cell recruitment to UPEC UTI, as caspase-1/11 KO mice are deficient in mast cell bladder recruitment (95). To complement mast cell-mediated inflammation, mast cells are the predominant producers of IL-10, suggesting dual roles of mast cells in initiating and resolving bladder inflammation (96). Mast cells and their associated proteases play a role in host protection against GBS in systemic and intrauterine infection (97) and in the context of vaginal colonization (98). GBS stimulates mast cell degranulation (98), but neither a prior study (98) nor this work (Fig. S5A) could demonstrate direct killing of GBS by the mast cell line HMC-1. It has been noted that this cell line is deficient in tryptase and chymase activity compared to the levels in mature human skin mast cells (99). Moreover, the contribution of mast cells to GBS UTI was not assessed using mast cell-deficient animals, which is a limitation of this study.

Diabetes mellitus and gestational diabetes are widely recognized risk factors for UTI (100, 101), including UTI caused by GBS (102, 103); however, the host immune deficiencies explaining this phenomenon are poorly understood. For instance, neutrophils from type 2 diabetics are equally as capable of GBS phagocytosis as those of healthy counterparts (104). Here, we introduce for the first time a murine diabetic model to study GBS UTI using multiple doses of streptozotocin (STZ) to induce necrosis of pancreatic beta cells (105). STZ-treated mice are more susceptible to UPEC UTI, in part through enhanced bacterial adherence (73). Although STZ treatment results in a model more like type 1 diabetes, similarities exist, including excessive glucosuria (Fig. 6). Recent reports of spontaneous GBS soft tissue infections in a type 2 diabetic genetic mouse model suggest that increased GBS susceptibility is maintained across multiple diabetic manifestations (106).

We found that STZ-treated mouse bladders had more GBS CFU, higher cathelicidin levels, and more infiltrating immune cells, making it difficult to distinguish immune populations impacted by host metabolic dysfunction and elevated responses to increased bacterial burdens. From these data, we cannot differentiate the source of cathelicidin in STZ-treated mice since bladder epithelial cells, as well as mast cells, neutrophils, and other myeloid lineage cells (which were increased in STZ-treated mice), are all known to produce cathelicidin. Higher cathelicidin in diabetes has been identified at inflamed mucosal cites (55), in the kidneys during acidosis (107), and in infected macrophages (108). Additionally, GBS burdens were no different between diabetic and nondiabetic *Camp*^−/−^ mice in our model (Fig. 7). Together, these studies implicate a detrimental impact of cathelicidin in control of GBS UTI in diabetes.

In both our genetic model of cathelicidin deficiency and the diabetic mouse model, increased bladder mast cells corresponded with higher GBS burdens. Mast cell degranulation promotes bladder epithelial cell shedding (95), and we speculate that this tissue damage response may enhance GBS adherence or invasion of the bladder. Cromolyn sodium reduces inflammation and reverses lower urinary tract dysfunction in a murine model of autoimmune cystitis (70) but, to our knowledge, has not been investigated in the context of UTI. Cromolyn sodium-mediated reduction of neutrophil chemotaxis and generation of oxygen radicals have been reported (109, 110). Although we did not observe changes in neutrophil myeloperoxidase in response to cromolyn sodium (Fig. 5C), involvement of other myeloid cells besides mast cells in explaining a reduction of GBS CFU levels in cromolyn-treated mice cannot be ruled out in this study. Our results using cromolyn sodium compellingly support the idea that inhibition of mast cells enhances host control of GBS in the bladder (Fig. 5 and 6). Cromolyn treatment lowered histamine levels and bacterial burdens in our GBS model in C57BL/6 mice (Fig. 5). However, in a CD1 mouse background, cromolyn sodium treatment did not alter histamine levels but did resolve bacterial burden differences between diabetic and nondiabetic animals (Fig. 7). These findings support a complex role for mast cells in control of UTI. Immune responses supported by mast cell degranulation, including IL-6 and tumor necrosis factor alpha (TNF-α), may impair neutrophil and macrophage bacterial clearance of GBS, as recently observed in Streptococcus pneumoniae lung infection (111), perhaps explaining why neutrophil MPO responses were higher in *Camp*^−/−^ mice (Fig. 4).

In summary, we have highlighted cathelicidin as a potent immune regulator, rather than an antimicrobial effector, in the bladder during GBS UTI, driving the recruitment of mast cells. There is mounting evidence for the importance of mast cells in modulating bladder function during both sterile and infection-mediated inflammation. In the context of GBS UTI, mast cells may be detrimental in controlling infection, and mechanistic insight is a focus of future studies. These findings expand our knowledge of the host factors contributing to GBS UTI and support cathelicidin and mast cell signaling pathways as targets for controlling GBS UTI in susceptible populations.

## MATERIALS AND METHODS

### Bacterial strains, growth conditions, and MIC and growth curve testing.

Group B *Streptococcus* (GBS) strains used in this study include COH1 (ATCC BAA-1176), A909 (ATCC BAA-1138), and NCTC 10/84 (ATCC 49447) and wild-type GBS clinical isolates from adults obtained from Sharp Memorial Hospital, San Diego, CA (IRB approval no. 1805802), which were grown for at least 16 h in stationary culture to stationary phase at 37°C in Todd-Hewitt Broth (THB) prior to experiments. Clinical GBS isolates were confirmed by growth as pink/mauve colonies on CHROMagar StrepB (catalog no. SB282; DRG International) as well as by the presence of the group B carbohydrate via latex bead agglutination (R30950701; Remel Streptex). For killing kinetics and MIC assays, overnight cultures were diluted 1:10 in fresh THB and incubated under stationary conditions at 37°C until mid-log phase (optical density at 600 nm [OD_600_] of 0.4).

Uropathogenic E. coli (UPEC) strain CFT073 (O6:K2:H1; ATCC 700928) was grown for at least 16 h in shaking culture to stationary phase at 37°C in Luria-Bertani (LB) broth prior to experiments. For MIC assays, overnight cultures were diluted 1:30 in fresh LB broth and incubated with shaking at 37°C until mid-log phase (OD_600_ of 0.4).

For killing or growth kinetics, log-phase GBS cultures were diluted 1:30 in RPMI 1640 medium (Gibco) in 96-well microtiter plates with or without 27 μg/ml (6 μM) or 36 μg/ml (8 μM) LL-37 (catalog no. 4042456; Bachem) in a 300-μl total volume. To test the effect of protease activity on GBS survival, Halt protease inhibitor cocktail (catalog no. PI78425, 1×; ThermoFisher), serine protease inhibitor cocktail (56-500-01VL, 1×; Millipore), (Z-LL)2 ketone cysteine protease inhibitor (42-105-05MG, 5 μM; Millipore), pepstatin A aspartyl protease inhibitor (50-114-6412, 10 μM; AdipoGen), and bestatin hydrochloride aminopeptidase inhibitor (B8385-1MG, 50 μM; Sigma-Aldrich) were added at the given concentrations. Plates were incubated under stationary conditions for 4 h at 37°C. At 0, 1, 2, and 4 h, samples were serially diluted and plated on THB agar. The number of CFU was determined and expressed as a percentage of the CFU count at time zero.

For MICs, mid-log-phase cultures were diluted 1:100 in synthetic urine (59) or RPMI 1640 medium (Gibco) with or without LL-37 or CRAMP (catalog no. 4056438; Bachem) at concentrations ranging from 0 to 288 μg/ml (0 to 64 μM) in a 100-μl total volume in 96-well microtiter plates. To test the effect of protease activity on MIC, Halt protease inhibitor cocktail (100×; ThermoFisher) was added at 1:100. Plates were incubated under stationary conditions for 24 h at 37°C. To measure metabolic activity, resazurin (Sigma-Aldrich) was added at 6.75 μg μg/ml, and plates were incubated at 37°C for 3 h. Fluorescence, indicated by resazurin-to-resofurin conversion, was read at an excitation wavelength of 550 nm and emission wavelength of 600 nm on an Enspire plate reader (Perkin-Elmer). The MIC was determined by a >90% reduction in fluorescent signal from the conversion of resazurin to fluorescent resorufin.

### Cell lines and infection assays.

The human bladder epithelial cell line 5637 (ATCC HTB-9) was cultured in RPMI 1640 medium (Gibco) supplemented with 10% heat-inactivated fetal bovine serum (FBS) at 37°C in humidified air with 5% CO_2_. The human mast cell line HMC-1 (112) was cultured in Iscove's modified Dulbecco's medium (IMDM) (catalog no. 21056-023; Gibco) supplemented with 1.2 mM 1-thioglycerol (M6145-100ML; Sigma-Aldrich) and 10% FBS at 37°C in humidified air with 5% CO_2_.

For LL-37 stimulation assays, HTB-9 cells were infected as described previously (44). Briefly, HTB-9 monolayers were grown in 24-well tissue culture plates to confluence. Fresh medium was added (400 μl), and cells were infected with log-phase GBS or UPEC in 100 μl at a multiplicity of infection (MOI) of 10 or 1 (bacterium-to-cell ratio). Cells were incubated for 3 h (qPCR) or 4 h (ELISA) at 37°C with 5% CO_2_. For transcript analyses, mRNA was extracted using a Purelink RNA minikit (ThermoFisher) per the manufacturer’s instructions. For protein analyses, cells were lysed with vigorous pipetting in radioimmunoprecipitation assay (RIPA) buffer and protease inhibitor cocktail, and samples were frozen at –20°C. Where applicable, 1× Halt protease inhibitor cocktail was added immediately prior to infection. For cell viability assays, supernatant was collected at the 4-h time point, and LDH was quantified using a CytoTox96 assay (Promega) per the manufacturer’s instructions. As a positive control, uninfected HTB-9 cells at the end of the 4 h of incubation were lysed with 1% Triton X-100. Data were expressed as percent lysis of the positive control.

For HMC-1 killing assays, cells were resuspended in fresh medium at 2 × 10^6^ cells/ml. Where applicable, HMC-1 cells were pretreated with 100 μM cromolyn sodium as described previously (72) at 37°C with 5% CO_2_ for 30 min. HMC-1 cells were seeded at 2 × 10^5^ cells/well in 96-well microtiter plates with or without 288 ng/ml (64 nM) LL-37. Log-phase COH1 or CFT073 was added at 2 × 10^4^ CFU in 10 μl for an MOI of 0.1. To facilitate cell-bacterium contact, plates were centrifuged at 300 × *g* for 5 min. Plates were incubated at 37°C with 5% CO_2_ for 60 min. Cells were lysed by vigorous pipetting and serial dilution in water and plated on THB agar. Percent killing was calculated as a percentage of the CFU total from wells containing bacteria only at the equivalent time point.

### qPCR.

HTB-9 mRNA was treated with a Turbo DNA-free kit (Invitrogen) per the manufacturer’s instructions. To synthesize cDNA, approximately 100 ng of total RNA was used with an iScript cDNA synthesis kit (Bio-Rad). cDNA was diluted 1:2 for real-time qPCR using Kapa SYBR qPCR 2× master mix (catalog no. KM4101; Kapa Biosystems) and performed on a Bio-Rad CFX96 real-time C1000 thermocycler. Primers were used at a final concentration of 200 nmol. Primer sequences used for quantification of human LL-37 mRNA were 5′-GAAGACCCAAAGGAATGGCC-3′ and 5′-CAGAGCCCAGAAGCCTGAGC-3′ as described previously (113). The relative transcript level was normalized to the level of the endogenous housekeeping gene GAPDH using the 2^−ΔΔ^*^CT^* (where *C_T_* is threshold cycle) method (114).

### Dot blots.

To assess GBS degradation of cathelicidin, stationary GBS cultures were diluted 1:10 in RPMI 1640 medium, and 100 μl per well was added to 96-well microtiter plates. LL-37 or CRAMP was added at 2 μM, a sub-MIC level. Cathelicidin standard curves (100 μl volume) at 36, 18, 9, 4.5, 2.3, and 1.1 μg/ml, as well as bacterium-only wells, were included as controls. As indicated in figure legends, to some wells, Halt protease inhibitor cocktail was included at a 1× final concentration. Samples were incubated at 37°C for 4 h and centrifuged at 3,220 × *g* for 5 min. Supernatant (5 μl) was spotted onto nitrocellulose membranes (catalog no. 162-0214; Bio-Rad) and allowed to dry completely. Membranes were blocked, with Odyssey blocking buffer diluted 1:1 with PBS for 1 h, with rocking at room temperature. Mouse anti-LL-37 (HM2071; Hycult) antibody or rabbit anti-CRAMP (pAb-antiCAMP/LL-37, NB100-98689; Novus) antibody was added at a 1:2,000 dilution and incubated, with rocking, for 2 h at room temperature. Membranes were washed three times with PBST (PBS with 0.1% Tween 20). Secondary goat anti-mouse antibody 680LT (926-68020; Li-Cor) or goat anti-rabbit IgG antibody 800CW (926-32211; Li-Cor) was added at 1:10,000 and incubated, with rocking, for 30 min at room temperature. Membranes were washed three times with PBST and two times with PBS and visualized on a Li-Cor Odyssey instrument. Spot intensity was measured using ImageJ software (NIH, version 1.52a) and normalized to the level of a 9-μg/ml LL-37 control.

### Animals.

Animal experiments were approved by the University of California (UC) San Diego Institutional Animal Care and Use Committee (IACUC) and conducted under accepted veterinary standards. Mice were allowed to eat and drink *ad libitum*. WT C57BL/6J male and female mice, aged 8 to 12 weeks, originally purchased from Jackson Laboratories, were bred in the same facility along with cathelicidin-deficient mice (*Camp*^−/−^) on the C57BL/6J background (115). Females were used for UTI and vaginal colonization studies, and males were used for GBS sepsis studies. WT CD1 female mice, 8 weeks of age, were purchased from Charles River Laboratories. For studies with exogenous CRAMP treatment, mice received 50 μl of CRAMP (diluted to 1.44 mg/ml in PBS, 72-μg total dose) transurethrally 1 h prior to infection, or control mice received 50 μl of PBS, as adapted from prior studies (52). To inhibit mast cell degranulation, mice were injected i.p. with cromolyn sodium (catalog no. C0399-1G; Sigma-Aldrich) at 10 mg/kg/dose in 100 μl at 48 h, 24 h, and 1 h prior to infection, as adapted from prior studies (70). Control mice were injected i.p. with 100 μl of PBS at each treatment time point. To induce diabetes mellitus, mice were injected i.p. with streptozotocin (catalog no. AG-CN2-0046-G001; Adipogen) at 80 mg/kg/dose in 200 μl of 0.1 M citrate buffer daily for 4 days. Control mice received 4 daily treatments of 200 μl of 0.1 M citrate buffer. Mice were weighed weekly thereafter. Blood glucose was determined 24 h prior to infection, and urinary glucose was determined at 24 h postinfection. Sample glucose was determined using an AimStrip Plus blood glucose meter kit.

### Murine urinary tract infection model.

An established mouse UTI protocol was used as previously described (44). Mice were anesthetized with inhaled isoflurane, and urine was voided from the bladder prior to transurethral infection with 100 μl of GBS COH1 or UPEC CFT073 (administered in ∼2 to 3 s) at 2 × 10^7^ CFU per mouse. Transurethral infection was achieved by inserting an UV-sterilized polyethylene tube (inner dimension, 0.28 mm; outer dimension, 0.61 mm) (catalog no. 598321; Harvard Apparatus) attached to a 30-gauge hypodermic needle into the urethra. At 24 h postinfection, urine samples were collected from each mouse prior to euthanasia and removal of bladders and both kidneys. For GBS CFU enumeration and ELISA, bladders and kidneys were homogenized in PBS containing silica beads (diameter, 1 mm; Biospec) and shaken at 6,000 rpm for 60 s using a MagNA Lyser (Roche). Homogenates were serially diluted and plated on THB agar.

### Murine vaginal colonization model.

Vaginal colonization studies were conducted as described previously (116). Briefly, mice were synchronized with 0.5 mg of β-estradiol administered i.p. 24 h prior to inoculation. Mice were inoculated with 10 μl (2 × 10^7^ CFU total) of GBS COH1 into the vaginal tract. The vaginal lumen was swabbed daily, and recovered GBS was quantified by plating on CHROMagar StrepB.

### Murine sepsis model.

For sepsis studies, mice were injected with GBS COH1 in 100 μl (2 × 10^7^ CFU) intraperitoneally as described previously (62). After 24 h, heparinized blood specimens were collected via cardiac puncture, and kidneys and spleens were homogenized in PBS containing silica beads (diameter, 1 mm) and shaken at 6,000 rpm for 60 s using a MagNA Lyser. Blood and tissue homogenates were plated on THB agar.

### Flow cytometry.

Bladder samples were subjected to flow cytometry as adapted from previous work (44, 117). Bladders were minced finely using a razor blade and then incubated in RPMI 1640 medium with 4 mg/ml collagenase and 50 U/ml DNase for 1 h at 37°C with pipetting every 15 min. Samples were passed through a 40-μm-pore-size filter, and cells were washed in RPMI 1640 medium with 10% FBS. Cells were blocked with 1:200 mouse BD Fc-block (BD Biosciences) for 15 min on ice in PBS with 1 mM EDTA, 1% FBS, and 0.1% sodium azide for 15 min on ice. Cells were stained for surface markers using the following antibodies at 5 μg/ml for 30 min on ice: anti-CD11b-fluorescein isothiocyanate (FITC) (clone M1/70, catalog no. 553310; BD Pharmingen), anti-c-kit-phycoerythrin (PE) (clone 2B8, catalog no. 1880-09; Southern Biotech), anti-CD11c-PerCP-Cy5.5 (where PerCp is peridinin chlorophyll protein) (clone N418, catalog no. 45-0114-82; eBioscience), anti-Ly6C-PE-Cy7 (clone HK1.4, catalog no. 25-5932-82; eBioscience), anti-Ly6G-allophycocyanin (APC) (clone 1A8, catalog no. 127614; BioLegend), anti-MHC-II-APC-Fire750 (clone M5/114.15.2, catalog no. 107652; BioLegend), anti-FcεRI-Pacific Blue (clone MAR-1, catalog no. 134313; BioLegend), and anti-CD45-BV510 (clone 30-F11, catalog no. 103138; BioLegend). Samples were gated on unstained cells, and positive signals were determined using single-stain controls. Samples were run on a BD FACSCanto II (BD Biosciences), and data were analyzed with FlowJo, version 10.2, software (FlowJo LLC).

### ELISA.

HTB-9 lysates were diluted 1:2 and assessed for LL-37 production using a human LL-37 ELISA kit (LS-F31974; LSBio) per the manufacturer’s instructions. Murine bladder homogenates were diluted 1:2 and subjected to CRAMP, histamine, and myeloperoxidase quantification using mouse cathelicidin (LS-F9642; LSBio), histamine (EA31; Oxford), and mouse MPO (DY3667; R&D Systems) ELISA kits per the manufacturers’ instructions.

### Statistics.

All data were collected from at least three biological replicates performed in at least technical duplicate as part of at least two independent experiments. When biological replicates were not available (e.g., immortalized cell lines and bacterium-only assays), experiments were performed independently at least three times. Mean values from technical replicates were used for statistical analyses, with independent experiment values or biological replicates represented in graphs with means ± standard errors of the means (SEM) or medians with 95% confidence intervals, as indicated in figure legends. All data sets were subjected to D’Agostino-Pearson normality test to determine whether values displayed Gaussian distribution before the appropriate parametric or nonparametric analyses were selected. In the instances where *in vitro*, *ex vivo*, and *in vivo* experimental numbers (*n*) were too small to determine normality, data were assumed to be nonparametric. The alpha level used for all tests was 0.05. HTB-9 cathelicidin ELISA values were analyzed by Friedman’s test with Dunn’s multiple-comparison test or by two-way analysis of variance (ANOVA) with Sidak’s multiple-comparison test, as indicated in the figure legends. GBS growth curves with LL-37 and protease inhibitors were analyzed using two-way repeated-measures ANOVA with Dunnett’s multiple-comparison test. MIC values for clinical isolates from different body sites and bladder cathelicidin ELISA of GBS- and UPEC-infected mice were analyzed using Kruskal-Wallis with Dunn’s multiple-comparison test. Tissue GBS CFU burdens, flow cytometry immune cell profiles, and mouse body weights were analyzed using two-way repeated-measures ANOVA with Sidak’s or Tukey’s multiple-comparison test, as indicated in the figure legends. Bladder histamine, cathelicidin, and MPO ELISAs were analyzed using one-way ANOVA with Holm-Sidak’s or Tukey’s multiple-comparison tests as indicated in the figure legends. Blood and urine glucose levels and bladder cathelicidin and histamine ELISA of STZ-treated mice were analyzed by two-tailed Mann-Whitney tests. Statistical analyses were performed using GraphPad Prism, version 7.03 (GraphPad Software Inc., La Jolla, CA). *P* values of <0.05 were considered statistically significant.
