# Supplementary figures and images for: Host Cathelicidin Exacerbates Group B Streptococcus Urinary Tract Infection
Source: mSphere. 2020 Apr 22;5(2):e00932-19. doi: 10.1128/mSphere.00932-19 (PMC7178553; doi:10.1128/mSphere.00932-19)

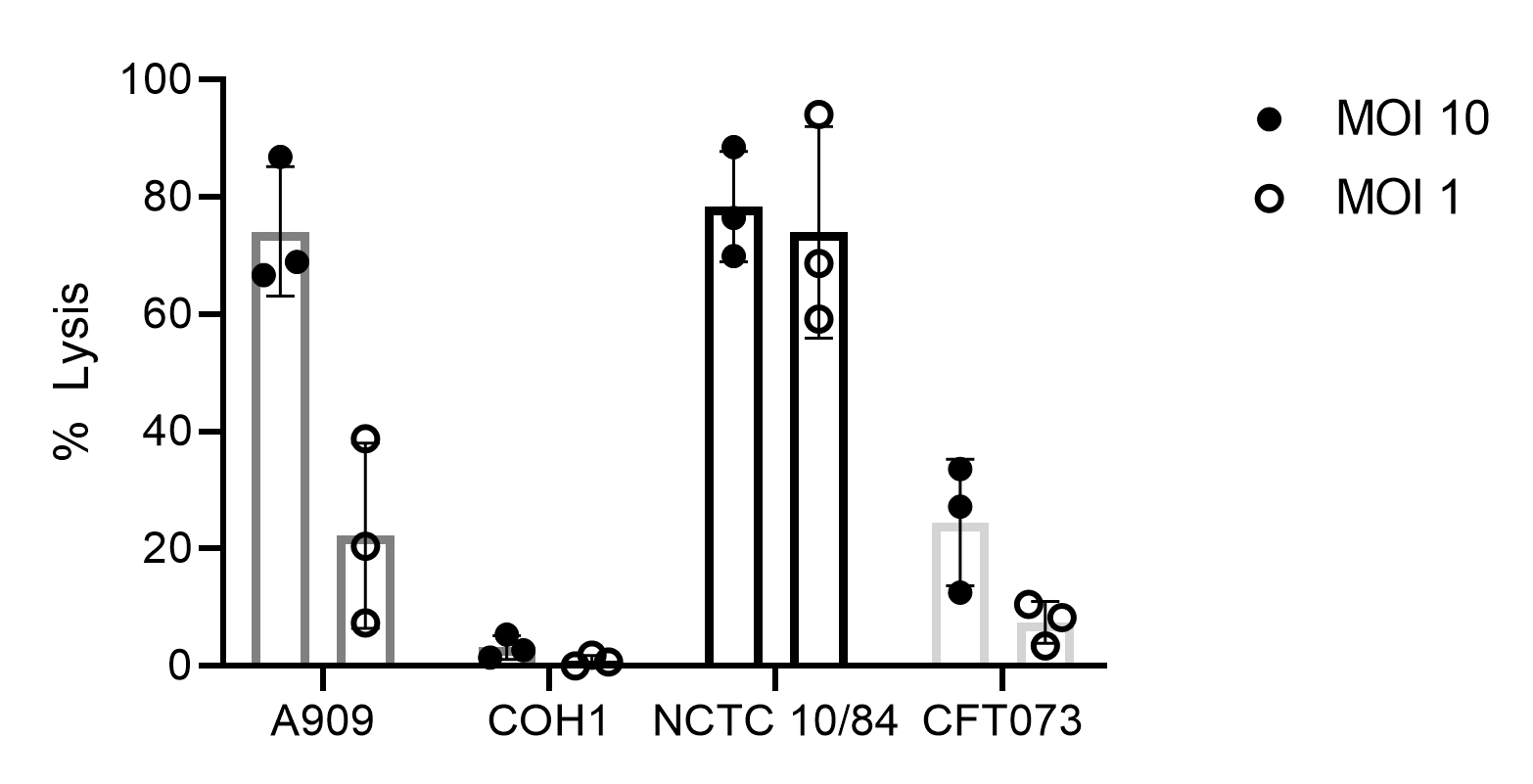

Supplement: FIG S1 [file mSphere.00932-19-sf001.tif]

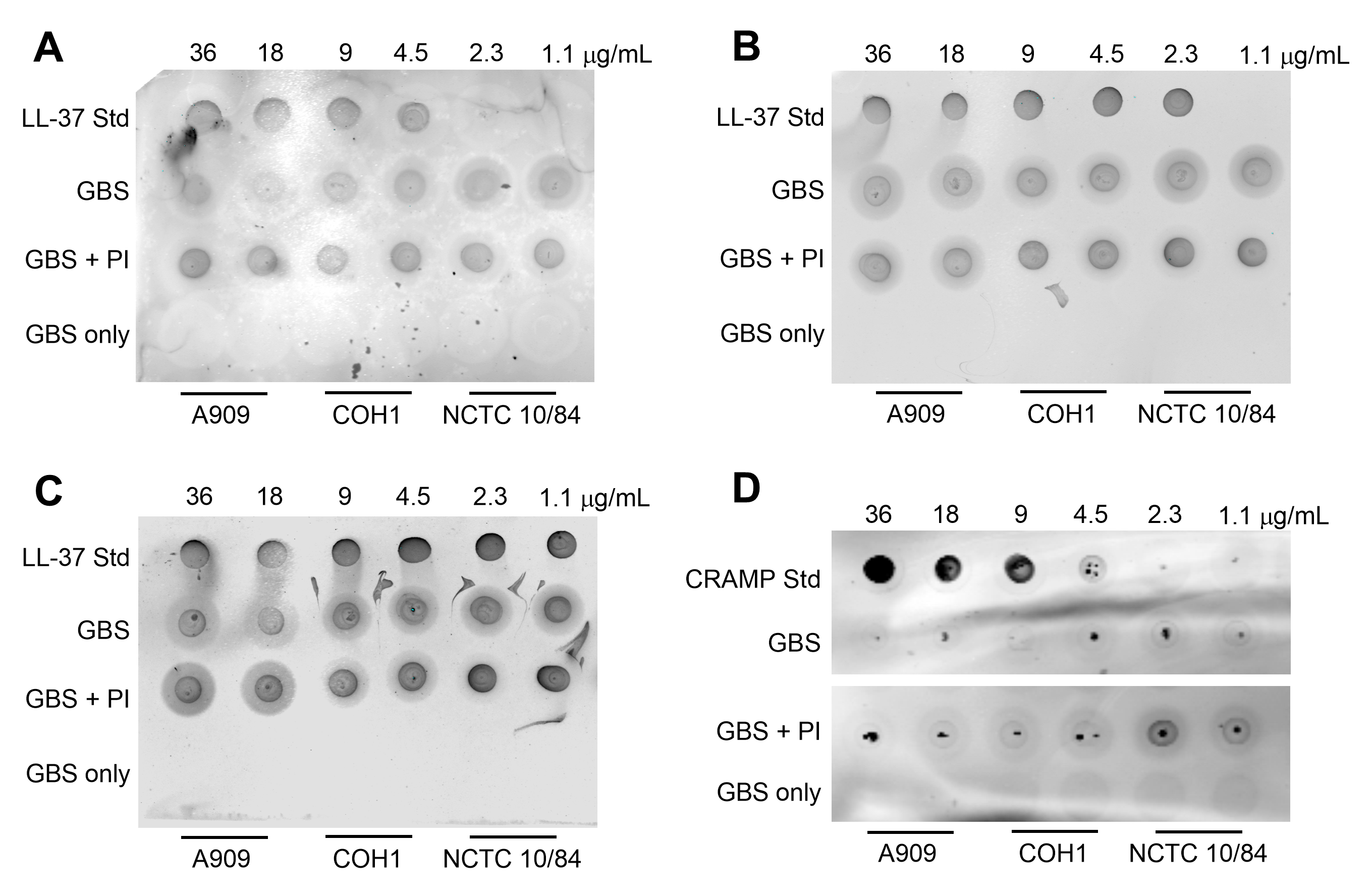

Supplement: FIG S2 [file mSphere.00932-19-sf002.tif]

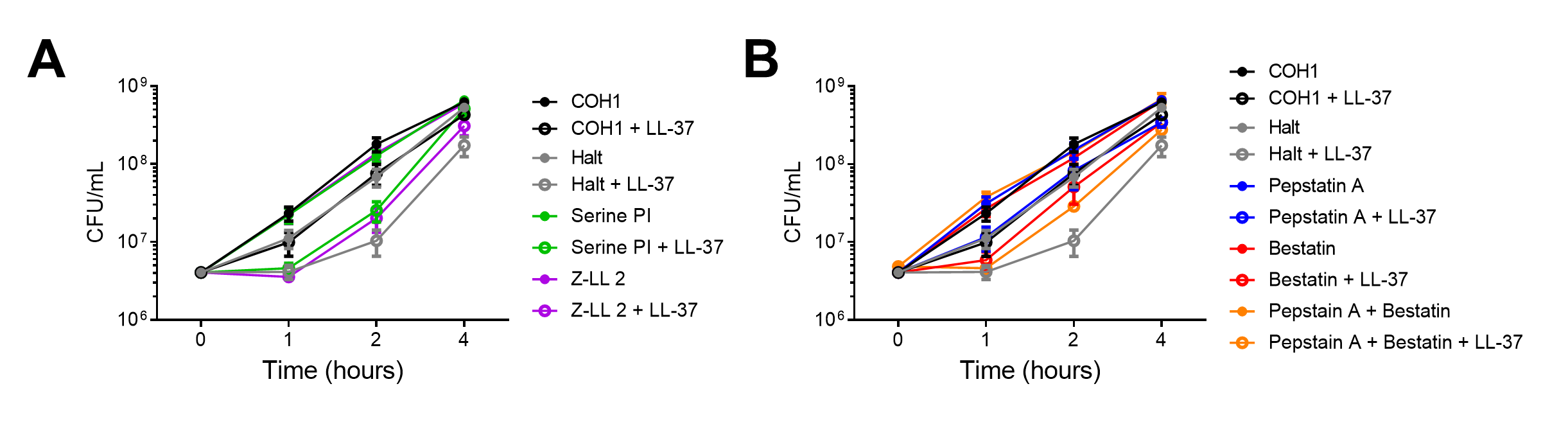

Supplement: FIG S3 [file mSphere.00932-19-sf003.tif]

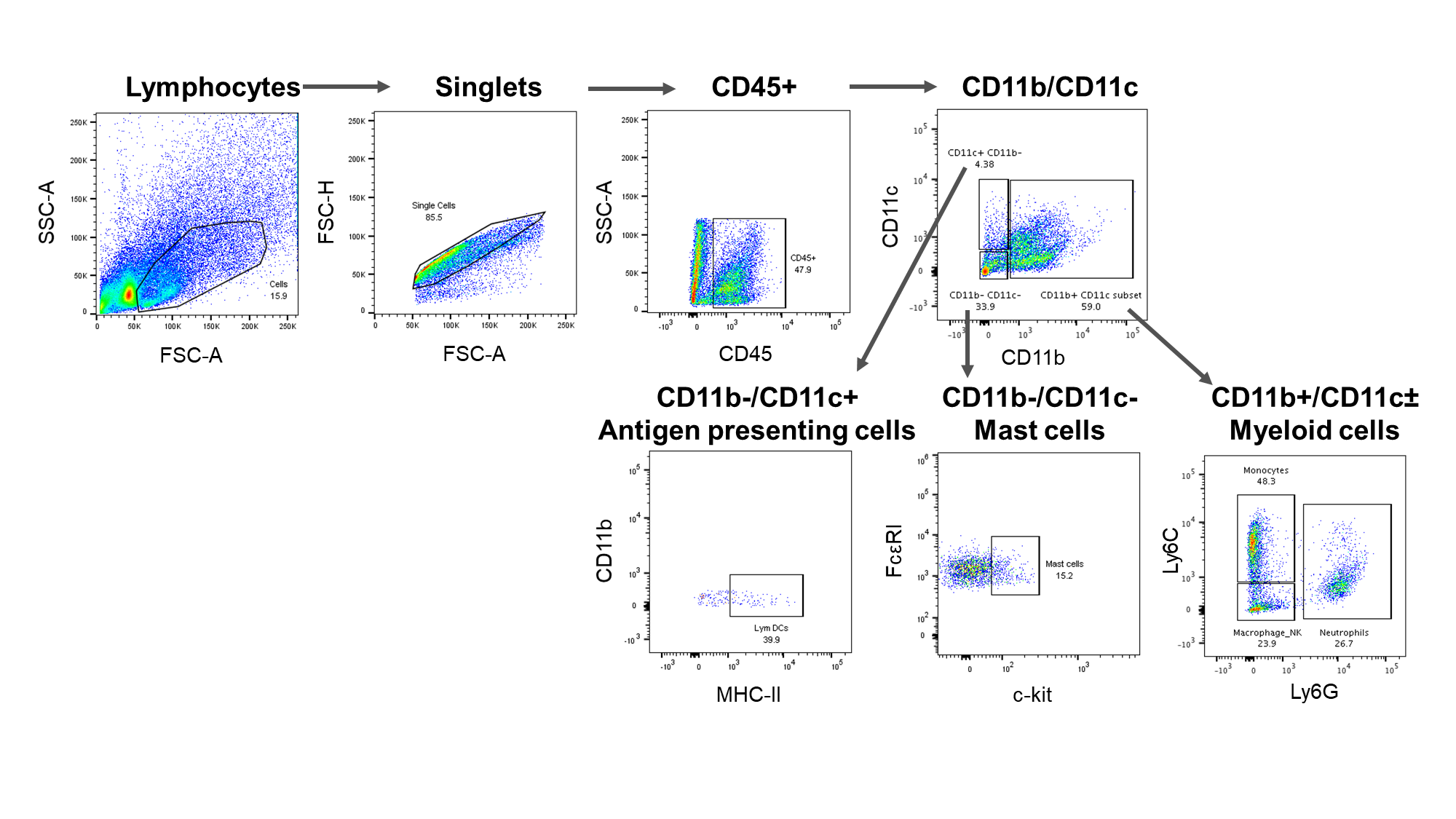

Supplement: FIG S4 [file mSphere.00932-19-sf004.tif]

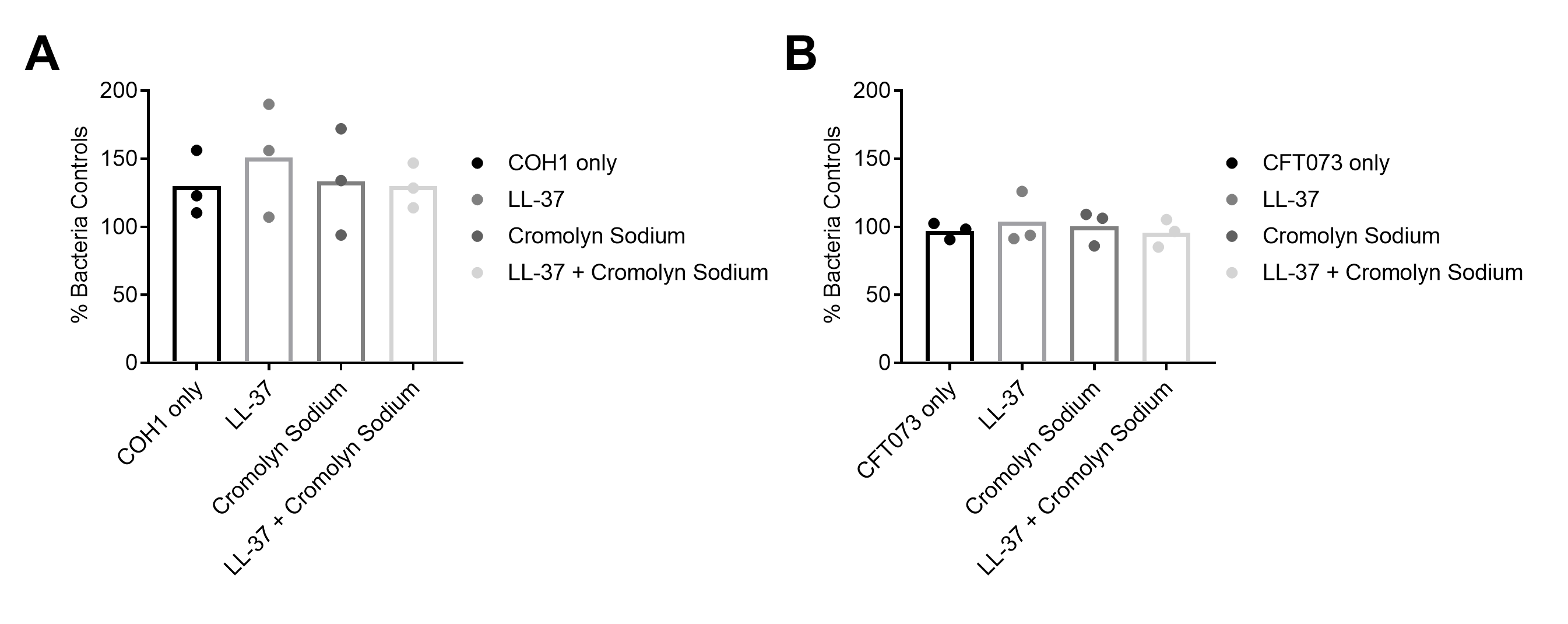

Supplement: FIG S5 [file mSphere.00932-19-sf005.tif]
